# Gamma knife knowledge‐based planning with isocenter selection

**DOI:** 10.1002/mp.70463

**Published:** 2026-05-14

**Authors:** Binghao Zhang, Mark Ruschin, Timothy C. Y. Chan

**Affiliations:** ^1^ Department of Mechanical and Industrial Engineering University of Toronto Toronto ON Canada; ^2^ Odette Cancer Centre Sunnybrook Health Sciences Centre Toronto Ontario Canada

**Keywords:** Gamma Knife, knowledge‐based planning, optimization

## Abstract

**Background:**

In Gamma Knife (GK) radiosurgery, effective treatment planning aims to achieve a desired dose distribution while minimizing the overall treatment duration. Clinically, plans are generated either manually through forward planning, or using inverse planning methods. To address the limitations presented by forward and inverse planning, a knowledge‐based planning (KBP) pipeline was recently developed for GK, using 3D dose predictions in combination with inverse optimization. However, this approach still relied on manually selected isocenter locations, which may limit plan quality.

**Purpose:**

To develop a complete KBP pipeline for GK, combining dose prediction with the simultaneous optimization of isocenter locations and beam‐on‐times.

**Methods:**

Data for 20 patients were obtained from a local care center. A previously trained deep learning model generated 3D dose predictions for each case. The primary treatment planning model was a mixed‐integer model (GK‐KBP‐OptIso) that combined isocenter selection with beam‐on‐time optimization. Plans were generated under several different maximum allowable isocenter limits and compared against (1) plans generated using an existing KBP pipeline with fixed, manually selected isocenters (GK‐KBP‐FixIso) and (2) historical clinical plans. Plan quality was assessed using coverage, selectivity, Paddick conformity, and gradient indices, along with overall treatment time.

**Results:**

Under the same isocenter limits, GK‐KBP‐OptIso achieved comparable conformity to GK‐KBP‐FixIso (0.657±0.161 vs 0.684±0.152) while significantly improving dose falloff. It also matched clinical plan quality, though with slightly increased treatment times. However, when allowed to use variable number of isocenters, GK‐KBP‐OptIso produced plans with a higher average conformity (0.717±0.124) than clinical plans (0.662±0.113).

**Conclusions:**

A KBP pipeline that integrates isocenter selection yields plans that are equal or superior to those generated using KBP with manually selected isocenters or manual clinical planning. The presented approach holds the potential to streamline the effort required to generate high‐quality GK treatment plans.

## 1 INTRODUCTION

Gamma Knife (GK) is a form of stereotactic radiosurgery used to treat brain tumors, arteriovenous malformations and various other neurological disorders.^1^ Effective GK treatment planning aims to produce plans that (1) deliver a dose distribution that achieves certain dosimetric objectives and (2) minimize beam‐on‐time and overall treatment duration.^2^

Two primary approaches are used for GK treatment planning: forward and inverse planning. In forward planning, isocenter locations, collimator combinations and beam‐on‐times are manually determined.^3^, ^4^ This is a time consuming process, requiring numerous iterations to achieve the desired dose distribution. Additionally, plan quality may vary with planner experience and time spent iterating during the planning process.^5^ In inverse planning, isocenter locations are first determined either algorithmically^6^, ^7^ or manually. The shots required to achieve a desired dose distribution are then determined using a multi‐objective optimization model, the objectives of which are based on select planning criteria.^8^ Each shot consists of a single dose of radiation, with a unique combination of isocenter location, collimator size and beam‐on‐time. With inverse planning, it may not be clear how to best balance competing objectives, and the weight for each objective is often manually determined.^9^

To streamline and improve consistency of the treatment planning process, other modalities such as intensity‐modulated radiotherapy (IMRT) and volumetric modulated arc therapy (VMAT), have adopted knowledge‐based planning (KBP),^10^, ^11^ which combines machine learning and optimization. By using information from historical plans, KBP can produce plans in an automated or semi‐automated manner, reducing reliance on planner experience and manual weight tuning. Recently, an analogous KBP approach was developed for GK,^12^ which combined deep learning‐based dose prediction^13^ with optimization to create a treatment plan. This approach, however, still relied on manually selecting isocenter locations. Isocenter selection significantly impacts plan quality, much like beam angle selection in IMRT/VMAT.^14^ Not only does isocenter selection potentially depend on planner experience, if done independently from the downstream plan optimization, a sub‐optimal plan may result. Previous work has explored the simultaneous optimization of isocenter locations and beam‐on‐time.^2^, ^7^ However, the methods were not used in a KBP framework.

In this paper, we develop a complete KBP pipeline for GK including (1) dose prediction, (2) isocenter location optimization, and (3) beam optimization. This pipeline's optimization model simultaneously optimizes isocenters and beams, resulting in a mixed‐integer programming model. To derive appropriate objective function weights for this model, an inverse integer optimization problem is solved. Note that inverse optimization^15^ is distinct from inverse planning. Inverse optimization is used to determine the weights to use in inverse planning, while inverse planning determines the actual shot delivery parameters.

We compare plan quality and treatment duration between KBP plans with optimized isocenters, KBP plans with manually selected isocenters, and historical clinical plans. The results demonstrate that isocenter optimization can be incorporated into KBP while maintaining plan quality.

## 2 METHODS

**FIGURE 1:**
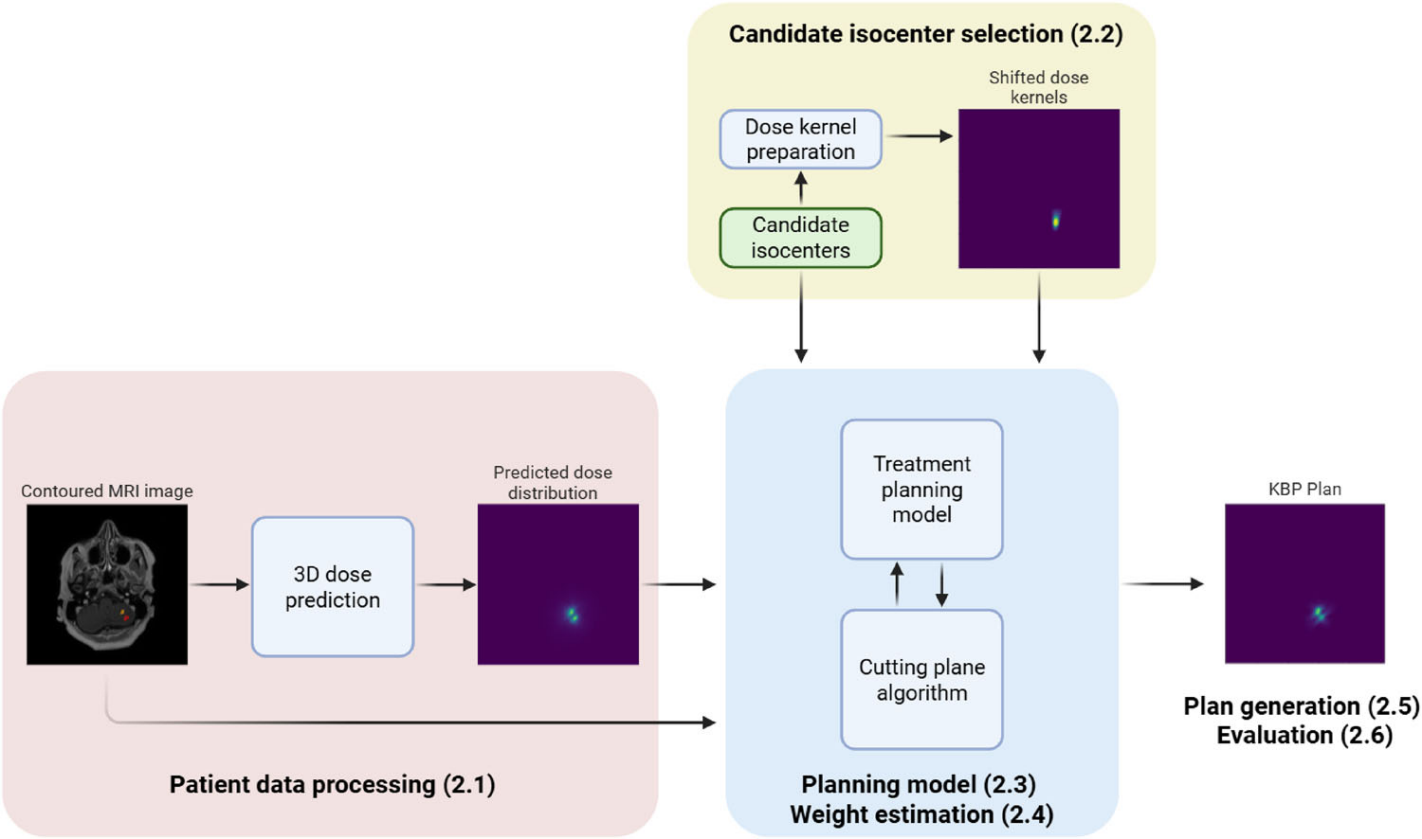
An overview of the planning pipeline. The main input to the pipeline is a contoured MRI image. In the prediction stage, denoted by red, the MRI is used to generate a 3D dose prediction. This prediction, along with a candidate set of isocenter locations is used in optimization stage, denoted by blue, where a cutting plane algorithm iterates with a treatment planning model to produce a final KBP treatment plan.

Figure 1 provides an overview of our methodology. Our approach comprises six steps: (2.1) patient data acquisition and dose prediction, (2.2) candidate isocenter identification, (2.3) treatment planning model formulation, (2.4) objective weight estimation using a cutting plane algorithm, (2.5) treatment plan generation, and (2.6) pipeline evaluation.

### 2.1 Patient data and dose prediction

Data from 20 patients treated for either brain metastases or intracranial schwannomas were obtained for this study. For each patient, MRI scans and delineated target contours were extracted and used as input into a previously developed deep learning model to generate 3D dose predictions.^13^ Historical clinical plans, created using manual forward planning, were also obtained for each patient to facilitate comparison. As no patients had organs‐at‐risk (OARs) in proximity to their targets, OAR sparing was not considered.

Our dataset contained target volumes ranging from 0.032 cc to 4.88 cc, with 32% of targets having volumes greater than 1.00 cc, and prescription doses ranging from 12.5 Gy to 25 Gy, with 85% prescribed either 18 or 20 Gy. Of the 20 patients, 8 had more than one target. Target sphericity ranged from 0.480 to 0.983, with an average value of 0.836 ± 0.10. Note that sphericity was normalized against a sphere of equal volume to account for the discrete nature of the voxel data. The original clinical plans had an average isocenter count of 5.89.

### 2.2 Candidate isocenter locations and dose kernels

To generate plans using optimization, each patient required a set of candidate isocenter locations and dose kernels.

We identified a set of candidate isocenter locations using an iterative sphere‐packing algorithm. For each target, 4mm‐diameter spheres were arranged in a hexagonal close‐packed structure within each target, with the sphere centroids designated as the isocenter locations. If the initial configuration did not yield a sufficient number of candidates (defined as at least 140% of the original clinical isocenter count or six candidate locations, whichever was greater), sphere diameters were reduced in 0.5mm increments until enough candidates were identified. To ensure balanced coverage for smaller targets, the centroid of each target was included as an additional candidate isocenter.

To test the impact of different candidate isocenter location selection methodologies, a different set of candidates was generated using a grassfire and sphere‐packing algorithm.^7^ We modified that algorithm to use spheres with diameters of 4, 3, and 2mm instead of 16, 8, and 4mm to ensure that enough candidate locations were generated to meet the criteria defined above.

Dose kernels provide a mapping from beam‐on‐time to dose for every sector and collimator combination at each isocenter location and are required to generate plans using optimization. Since the optimized isocenter locations are unknown in advance, a general dose kernel central to each patient's skull was generated using the GammaPlan TMR10 algorithm.^16^ After potential isocenter locations were determined following the methods above, the central kernel was translated to each candidate location to approximate location‐specific kernels, with voxels outside the skull assigned zero dose. Previous validation^12^ showed that these approximate kernels produced KBP plans with similar dose profiles and minimal dose differences compared to plans created using fully calculated TMR10 kernels.

### 2.3 Treatment planning model

Our primary treatment planning optimization model was a mixed‐integer model that combined isocenter selection with beam time optimization (GK‐KBP‐OptIso). The beam optimization part of the model was based on an existing inverse planning model that produces treatment plans optimized for a weighted objective given fixed isocenter locations (GK‐KBP‐FixIso).^8^, ^12^ The mathematical formulation for GK‐KBP‐OptIso was:

(1) $$\begin{array}{cc} \mathrm{minimize} & \;\sum_{p=1}^{P}(\frac{w_{T_{\min}p}}{D_{Tp}N_{Tp}}\sum_{j\in N_{Tp}}\max\left(D_{Tp}-f_{j}\left(t\right),0\right)\\ & +\;\frac{w_{T_{\max}p}}{2D_{Tp}N_{Tp}}\sum_{j\in N_{Tp}}\max\left(f_{j}\left(t\right)-2D_{Tp},0\right)\\ & +\;\frac{w_{Sp}}{D_{Sp}N_{Sp}}\sum_{k\in N_{Sp}}\max\left(f_{k}\left(t\right)-D_{Sp},0\right)\\ & +\;\frac{w_{Gp}}{D_{Gp}N_{Gp}}\sum_{l\in N_{Gp}}\max\left(f_{l}\left(t\right)-D_{Gp},0\right)\\ & \;+\frac{w_{\text{BOT}p}}{\frac{D_{Tp}}{D_{\text{cal}}}}\tau_{p}^{i\text{BOT}})\\ \mathrm{subject}\mathrm{to} & \;f_{r}\left(t\right)=\sum_{i=1}^{N_{I}}\sum_{s=1}^{8}\sum_{c=1}^{3}\phi_{risc}t_{isc},\\ & \;\forall r\in N_{Tp}\cup N_{Sp}\cup N_{Gp},\;p\in P\\ & \;\tau_{p}^{i\text{BOT}}=\sum_{i\in N_{Ip}}\max_{s=1,\cdots ,8}\sum_{c=1}^{3}t_{isc}\\ & \;\sum_{i\in N_{Ip}}z_{i}\leq H_{p},\;\forall p\in P\\ & \;z_{i}M\geq t_{isc},\;\forall i\in N_{I},\;s=\left\{1,2\text{…},8\right\},\;c=\left\{1,2,3\right\}\\ & \;t_{isc}\geq 0,\;\forall i\in N_{I},\;s=\left\{1,2\text{…},8\right\},\;c=\left\{1,2,3\right\}\\ & \;z\in \{0,1\}. \end{array}$$

In this model, P denotes the set of targets for a given patient, with $H_{p}$ specifying the maximum number of isocenters for each target p∈P. The candidate isocenter locations in target p is $N_{I_{p}}$ and $N_{I}=\cup_{p\in P}N_{I_{p}}$, the set of all candidate locations across all targets. A binary variable $z_{i}$ indicates whether an isocenter $i\in N_{I}$ is selected.

The decision variables are the beam‐on‐times, $t_{isc}$, defined for every combination of isocenter i, sector s, and collimator size c. The rate of dose contribution of each beam is represented by the dose kernel ϕ, with the total amount of dose delivered to each voxel r denoted by $f_{r}\left(t\right)$.

The model objective function contains five components which control different aspects related to plan quality: (1) target under‐dose, relative to a threshold of $D_{T}$, (2) target over‐dose, with threshold of 2$D_{T}$, (3) over‐dose immediately outside the target, with threshold $D_{S}$, (4) dose falloff, with threshold $D_{G}$, (5) beam‐on‐time, penalized by $\tau^{i\text{BOT}}$. The total objective function was the sum of these objectives over all targets. The objectives had associated weights, $w_{T_{\min}}$, $w_{T_{\max}}$, $w_{S}$, $w_{G}$, $w_{\text{BOT}}$, reflecting their relative importance. Each objective is normalized by its associated dose threshold, except for the beam‐on‐time objective which is instead scaled by a ratio involving a calibration dose, $D_{\text{cal}}$. The optimization is carried out over target volumes $N_{T}$, $N_{S}$, $N_{G}$, with the number of voxels in each volume being represented by $N_{T}$, $N_{S}$, and $N_{G}$, respectively.

### 2.4 Objective weight estimation

An inverse optimization model was developed to estimate the objective function weights for GK‐KBP‐OptIso, given a 3D dose prediction as input. Due to the size of the mixed‐integer program and the inherent noise in dose predictions, existing methods for solving the inverse optimization problem exactly^17^, ^18^ were not applicable.

Instead, we formulated an inverse optimization model based on the linear relaxation of (1). This model optimizes the relative duality gap^19^ associated with the predicted dose distribution and is formulated following the standard duality approach applied to a multi‐objective linear program^20^:

(2) $$\begin{array}{cc} & \mathrm{minimize}\;\sum_{p=1}^{P}(\frac{w_{T_{\min}p}}{D_{Tp}N_{Tp}}\sum_{j\in N_{Tp}}{\hat{y}}_{Tj}^{-}\\ & \;+\frac{w_{T_{\max}p}}{2D_{Tp}N_{Tp}}\sum_{j\in N_{Tp}}{\hat{y}}_{Mj}^{+}+\frac{w_{Sp}}{D_{Sp}N_{Sp}}\sum_{k\in N_{Sp}}{\hat{y}}_{Sk}^{+}\\ & \;+\frac{w_{Gp}}{D_{Gp}N_{Gp}}\sum_{l\in N_{Gp}}{\hat{y}}_{Gl}^{+}+\frac{w_{\text{BOT}p}}{\frac{D_{Tp}}{D_{\text{cal}}}}{\hat{\tau }}_{p})\\ & \mathrm{subject}\mathrm{to}\;\sum_{j=1}^{N_{T}}\phi_{jisc}q_{Tj}+\sum_{j=1}^{N_{T}}\phi_{jisc}q_{Mj}+\sum_{k=1}^{N_{S}}\phi_{kisc}q_{Sk}\\ & \;+\sum_{l=1}^{N_{G}}\phi_{lisc}q_{Gl}+q_{\text{BOT}is}+q_{Zi}\leq 0\\ & \;\sum_{p=1}^{P}\left(D_{Tp}\sum_{j=1}^{N_{Tp}}q_{Tj}+2D_{Tp}\sum_{j=1}^{N_{Tp}}q_{Mj}+D_{Sp}\sum_{k=1}^{N_{Sp}}q_{Sk}\right.\\ & \;\left.+D_{Gp}\sum_{l=1}^{N_{Gp}}q_{Gl}+H_{p}q_{H}\right)=1\\ & \;q_{H}-Mq_{Zi}\leq 0,\;\forall i\in N_{Ip},\;p\in P\\ & \;0\leq q_{Tj}\leq \frac{w_{T_{\min}p}}{D_{Tp}N_{Tp}},\;\forall j\in N_{Tp},\;p\in P\\ & \;0\geq q_{Mj}\geq -\frac{w_{T_{\max}p}}{2D_{Tp}N_{Tp}},\;\forall j\in N_{Tp},\;p\in P\\ & \;0\geq q_{Sk}\geq -\frac{w_{Sp}}{D_{Sp}N_{Sp}},\;\forall k\in N_{Sp},\;p\in P\\ & \;0\geq q_{Gl}\geq -\frac{w_{Gp}}{D_{Gp}N_{Gp}},\;\forall l\in N_{Gp},\;p\in P\\ & \;q_{\text{BOT}is}\leq 0,\;\forall i\in N_{Ip},\;s\in \left\{1,2\text{…},8\right\},\;p\in P\\ & \;\sum_{s=1}^{8}q_{\text{BOT}is}\geq -\frac{w_{\text{BOT}}}{\frac{D_{Tp}}{D_{\text{cal}}}},\;\forall i\in N_{Ip},\;s\in \left\{1,2,\text{…},8\right\},\;p\in P\\ & \;0\geq q_{Z},q_{H}\\ & \;w_{T_{\min}},w_{T_{\max}},w_{S},w_{G},w_{\text{BOT}}\geq 0. \end{array}$$

In this formulation, the q variables denote the dual variables associated with the constraints of (1). The dose prediction is represented using linear penalties, parameterized by ${\hat{y}}_{T}^{-}$, ${\hat{y}}_{M}^{+}$, ${\hat{y}}_{S}^{+}$, ${\hat{y}}_{G}^{+}$. The output of this model are the weight values $w_{T_{\min}}$, $w_{T_{\max}}$, $w_{S}$, $w_{G}$, $w_{\text{BOT}}$ that minimize sub‐optimality of the predicted dose distribution as measured by the relaxed problem.

Because these weights are optimized for the relaxed problem and not (1), which is our goal, these weights are not the final inverse planning objective weights. Rather, they are used to initialize a cutting plane algorithm,^21^ which iteratively refines these estimates to determine the final objective weights. The cutting plane algorithm iteratively alternates between (1) solving a master problem that minimizes the sub‐optimality gap over a set of feasible solutions to Equation (1) and produces an updated set of weights, and (2) generating new solutions to Equation (1) using the updated weights. This process refines the weight estimates until no further reduction in the sub‐optimality gap is possible, yielding weights that guarantee the predicted dose distribution is as close to optimal as possible. The algorithm is guaranteed to terminate in a finite number of iterations.^21^

To speed up the algorithm and reduce the number of iterations needed, an alternate approach involving regularization was also tested. To ensure the cutting plane algorithm generates weight vectors leading to meaningful solutions at every iteration, we incorporated regularization terms into the objective function of the master problem by imposing an $\ell_{1}$‐norm penalty. This penalizes solutions that deviate from the current best weight vector. Since the initial objective weights obtained from the inverse of the linear relaxation yield a valid dose close to the prediction, subsequent solutions will also be of high‐quality, and a high‐quality terminal solution can be reached in fewer iterations. The regularization is controlled using a λ parameter that is set to 10% of the current sub‐optimality gap.

### 2.5 Plan generation

**FIGURE 2:**
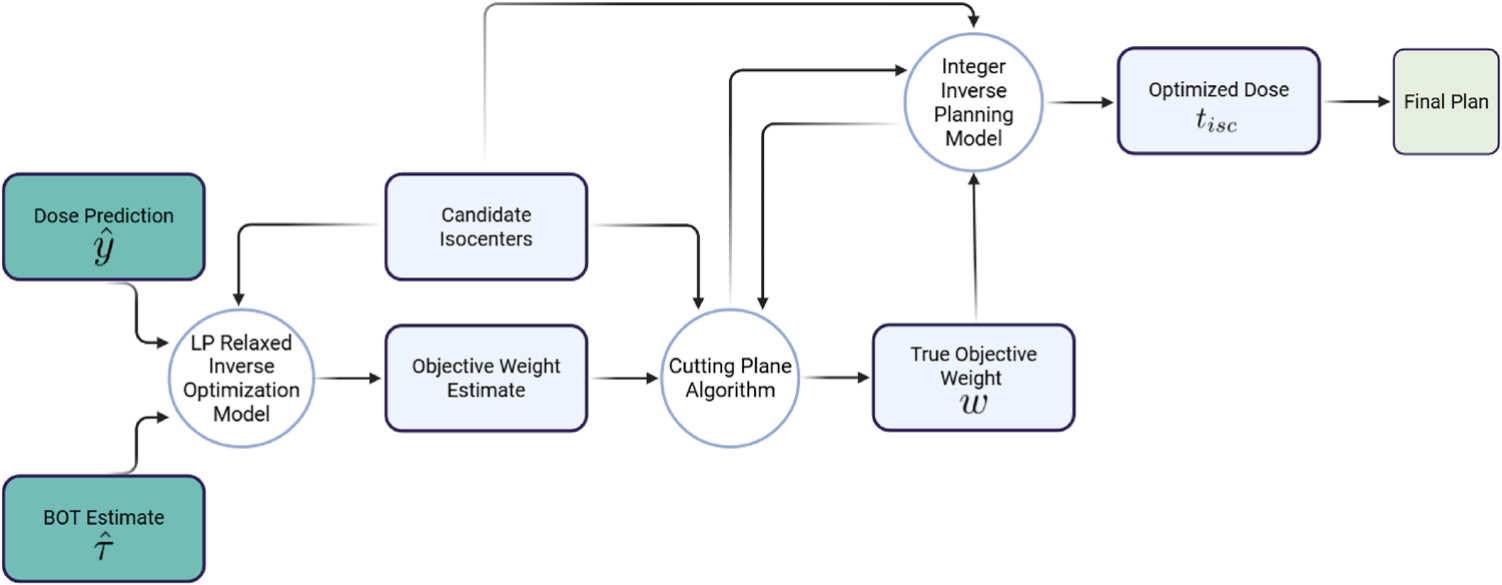
An overview of inputs and outputs in GK‐KBP‐OptIso. Compared to GK‐KBP‐FixIso, this requires the identification of candidate isocenters, and an additional cutting plan algorithm to calculate an optimal dose and generate the final plan.

The patient data and dose kernels were used in GK‐KBP‐OptIso to generate new treatment plans. The optimization models were solved using Gurobi 9.0.1 ^22^ with default parameters using two computers with i7‐12900K CPUs. The pipeline is illustrated in Figure 2.

The plan generation process begins by inputting the predicted dose distribution, a beam‐on‐time estimate (described below), candidate isocenter locations, and maximum allowable isocenters into formulation (2). This produces a set of objective weights to initialize the cutting plane algorithm. The final weights produced by this algorithm are used in the mixed‐integer inverse planning model (1) to calculate optimal beam‐on‐times. Finally, shots are generated using the calculated beam‐on‐times using a previous developed optimization model,^12^ allowing for plan delivery comparisons.

The cutting plane algorithm assumes the known solution, the prediction in this case, is feasible for the integer problem (1) or its linearly relaxed version. In our case, this assumption will be met given: (1) there are enough candidate isocenter locations identified, (2) a clinically appropriate isocenter limit is provided, and (3) the dose prediction is of sufficient quality. If the assumption is not met (e.g., overly restrictive isocenter limits), the cutting plane algorithm terminates immediately and the pipeline defaults to the weights obtained from formulation (2). The pipeline also defaults to these weights if a patient has exceptionally large targets where the computation time of the cutting plane algorithm exceeds one hour. To derive the beam‐on‐time estimates, we used a previously developed linear regression model formulated based on clinical plans.^12^ The estimate of the time, in minutes, needed to realize the dose prediction for a target p, given the prescription dose $D_{Tp}$, target volume $V_{p}$, and desired number of isocenters $N_{Ip}$ is

(3) $$\begin{array}{cc} & {\hat{\tau }}_{p}^{i\text{BOT}}=-0.3663D_{Tp}-9.595V_{p}+3.438N_{Ip}+14.94. \end{array}$$

### 2.6 Pipeline evaluation

We generated plans using GK‐KBP‐OptIso for all patients under three different levels of maximum allowable isocenters, termed isocenter limits (I). These limits were based on the corresponding clinical plan: 60% ($I_{0}$), 100% ($I_{1}$), and 140% ($I_{2}$). For each patient, a subjective “best” plan was selected across $I_{0}$, $I_{1}$, $I_{2}$. A plan was considered superior if it achieved at least a 3% improvement in conformity without a significant increase in treatment time (≤ 3 min), or a reduction in treatment time of at least 3 minutes without a significant loss in conformity (≤ 3%). Parallel plans were also generated using GK‐KBP‐FixIso pipeline, using the same beam‐on‐time estimates.

To evaluate the performance of the pipeline without relying on isocenter limits derived from prior clinical plans, we also used a predicted isocenter limit ($I_{\text{pred}}$). This limit was estimated based on target volume (V) and prescription dose ($D_{T}$) using a third‐degree polynomial ($R^{2}$ = 0.731)

(4) $$\begin{array}{cc} I_{\text{pred}} & =0.0024{D_{T}}^{3}-0.0843{D_{T}}^{2}-0.0052D_{T}\\ & \;+0.0009V+17.354 \end{array}$$

trained on data from 272 patients obtained from a previous study.^13^

To compare candidate location selection methodologies, additional plans were generated under $I_{1}$ using candidates obtained from the grassfire and sphere‐packing algorithm.

Plan quality was assessed using four voxel‐based metrics: coverage, selectivity, Paddick conformity, and gradient indices.^23^ The overall treatment time of each plan was also calculated and compared. Significance testing was done using the Wilcoxon signed‐rank test.

## 3 RESULTS

### 3.1 Dose quality and treatment time

**FIGURE 3:**
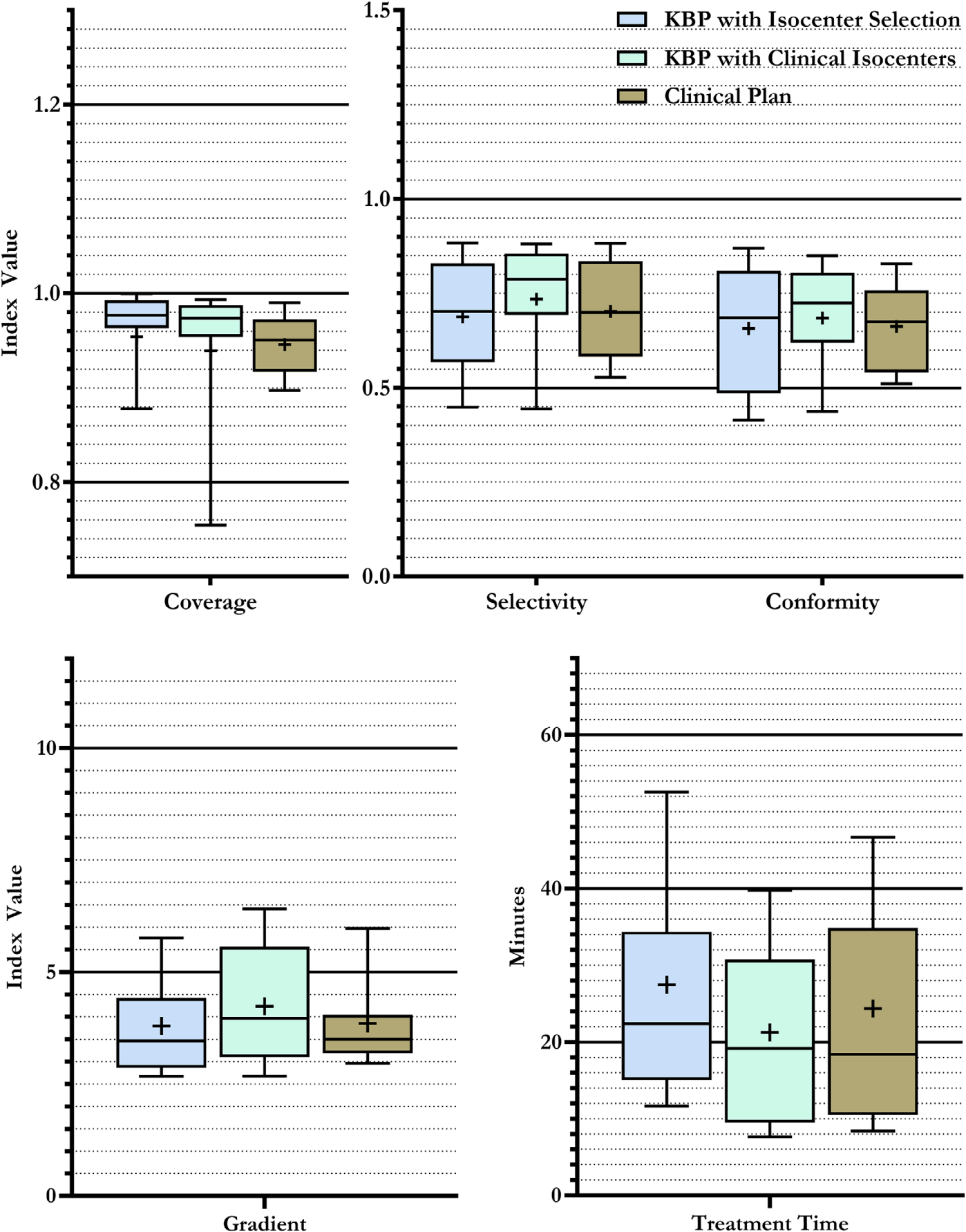
Comparison of quality metrics for KBP plans with algorithmically selected isocenters (GK‐KBP‐OptIso), KBP plans with manual isocenters (GK‐KBP‐FixIso), and clinical plans.

Figure 3 compares the distribution of the plan quality metrics for three types of plans: (1) plans generated using GK‐KBP‐OptIso, (2) plans generated using GK‐KBP‐FixIso using the clinical isocenters, and (3) clinical plans. All plans had the same isocenter limit, $I_{1}$. GK‐KBP‐OptIso achieved comparable conformity to GK‐KBP‐FixIso (0.657±0.161 vs. 0.684±0.152; W = 147, p = 0.210) while improving dose falloff, as seen by the lower average gradient index (3.80±1.14 vs. 4.24±1.37; W = 98, p = 0.016). GK‐KBP‐OptIso matched the overall quality of the clinical plans, though at the cost of a slightly higher treatment times (W = 28, *p* < 0.01).

Table 1 presents the mean and standard deviation of the plan quality metrics for the subjectively selected best plans, generated using GK‐KBP‐OptIso across isocenter limits of $I_{0}$, $I_{1}$, $I_{2}$. The best plans had significantly higher quality compared to both the GK‐KBP‐FixIso plans and clinical plans. However, these plans typically required more isocenters, leading to higher average treatment times.

**TABLE 1:** Mean and standard deviation in coverage index, selectivity index, conformity index, gradient index, and treatment time across three planning strategies: KBP with algorithmic isocenters, KBP with manual isocenters, and clinical plans. The “best” KBP plans with algorithmic isocenters were subjectively chosen between plans resulting from isocenter limits $I_{0}$, $I_{1}$, and $I_{2}$, and had generally higher isocenter counts. Adjusted treatment time incorporates an estimated 8 s delay when transitioning from each isocenter.

Metric	Best of GK‐KBP‐OptIso	GK‐KBP‐FixIso	Clinical
	*		
Coverage	0.959±0.078	0.939±0.093	0.946±0.035
Selectivity	0.747±0.111	0.734±0.166	0.702±0.131
Conformity	0.717±0.124	0.684±0.152	0.662±0.113
	*		
Gradient	3.86±1.20	4.24±1.37	3.86±1.00
Treatment	*		
time (min)	27.2±10.1	21.3±12.1	24.4±17.9
Adj. treat	*		
time (min)	28.2±10.9	22.0±12.7	25.2±18.6

*Note*: Significant differences compared to GK‐KBP‐FixIso are denoted by *.

### 3.2 Isocenter limits

**FIGURE 4:**
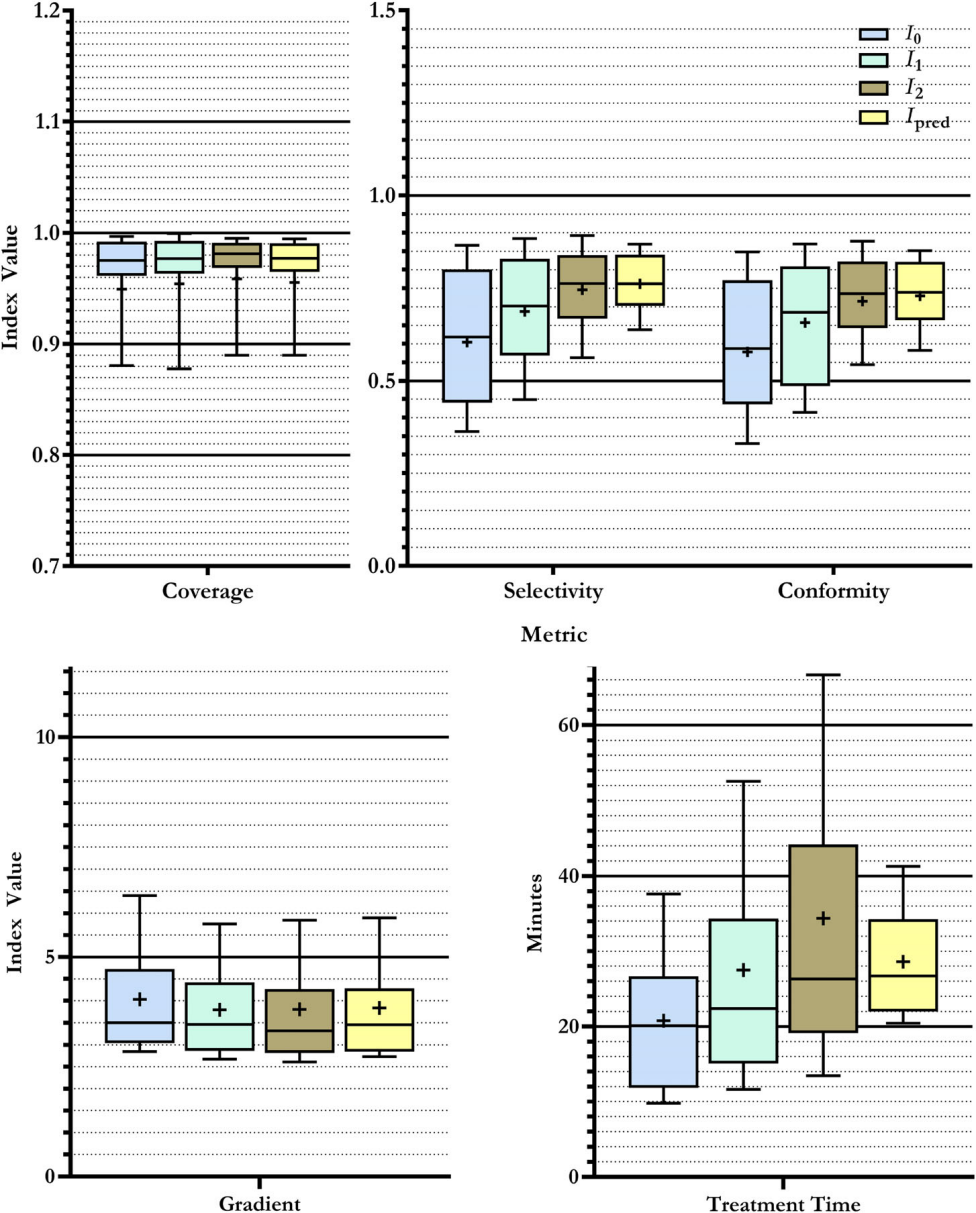
Comparison of quality metrics for KBP plans with generated using the modified pipeline, under four different isocenter limits, I. $I_{0}$ (60%), $I_{1}$ (100%), and $I_{2}$ (140%) are limits based on the corresponding clinical plan. $I_{\text{pred}}$ is a limit estimated using the features of the target based on other historical plans.

Figure 4 illustrates the relationship between the number of allowable isocenters and plan quality. As seen, increasing the isocenter limit from $I_{0}$ to $I_{2}$ significantly improved conformity (0.579±0.193 compared to 0.715±0.122). However, this comes at the cost of extending the overall treatment duration.

Notably, plans generated with an isocenter limit of $I_{\text{pred}}$ exhibited higher average conformity (0.729±0.111) compared to those generated with the isocenter limit used in the original clinical plans, $I_{1}$ (0.657±0.161), while maintaining similar treatment times to the clinical plan. Plans generated with an isocenter limit of $I_{\text{pred}}$ have, on average, more isocenters compared with the $I_{1}$, with a median difference of 1.5.

### 3.3 Impact of candidate selection

Table 2 compares the quality of the plans generated using the two different candidate isocenter selection methodologies under the isocenter limit of $I_{1}$. The differences in plan quality were small and statistically insignificant, with our iterative sphere‐packing algorithm producing plans of slightly higher quality compared to the combined grassfire and sphere‐packing algorithm.

**TABLE 2:** Quality metrics for KBP plans generated using two sets of candidate locations under $I_{1}$. There were no statistically significant differences in plan quality under different candidate selection methodologies.

Metric	Iterative sphere‐packing	Grassfire and sphere‐packing
Coverage	0.954±0.084	0.952±0.082
Selectivity	0.687±0.153	0.659±0.156
Conformity	0.657±0.164	0.625±0.154
Gradient	3.80±1.16	4.22±1.56
Treatment time (min)	27.5±15.7	26.3±14.9

### 3.4 Approximate weights

Table 3 evaluates the quality of plans generated using the three weight determination methods: (1) cutting plane algorithm, (2) cutting plane algorithm with regularization, and (3) solving formulation (2) only (i.e., the weights that were used to initialize the cutting plane algorithm, which we refer to as “approximate weights”).

**TABLE 3:** Mean and standard deviation in coverage index, selectivity index, conformity index, gradient index, and treatment time across three objective weight calculations: cutting plane algorithm, cutting plane algorithm with regularization, and a relaxed approximation resulting from solving formulation (2). No statistical difference exists when comparing the cutting plane approach to the relaxed approximation.

Metric	Cutting plane	Cutting plane (regularization)	Relaxed approximation
Coverage	0.946±0.091	0.942±0.090	0.948±0.086
Selectivity	0.705±0.159	0.711±0.153	0.707±0.152
Conformity	0.669±0.174	0.673±0.170	0.673±0.166
Gradient	3.84±1.26	4.22±2.11	3.89±1.68
Treatment			
time (min)	30.4±15.9	30.6±15.8	29.6±16.2
Cuts			
generated	15.8±7.45	8.50±5.10	N/A

The results demonstrate that plans generated using the approximate weights were very similar to those generated using the full cutting plane algorithm, but with a slightly higher duality gap (5.3%). The cutting plane algorithm was solved for the simplest targets in under 1 minute, with an average solution time of 12 min. The most complex targets exceeded our one hour threshold and were excluded from this average. Using the relaxed approximation, the most complex target was solved in 37 min.

Applying regularization to the cutting plane algorithm also did not appear to significantly affect the quality of the resulting plans. However, the computational efficiency of the algorithm was improved, with the average number of iterations being cut nearly in half.

## 4 DISCUSSION

This study introduces the first KBP pipeline for GK that simultaneously optimizes both the beam‐on‐times and isocenter locations. Our results demonstrate that, for the same number of isocenters, this KBP methodology produces plans of non‐inferior quality compared to both KBP and clinical plans generated using manually selected isocenters. In other words, isocenter selection has the potential to be integrated into KBP methods for GK while maintaining plan quality. While inverse planning with isocenter selection has been studied previously for GK,^2^, ^7^ this is the first study to integrate it in a KBP framework. Additionally, prior inverse planning models incorporating isocenter selection did not control for dose falloff as part of their objective,^2^ focusing only on tumor coverage and OAR sparing.

Under the isocenter limit $I_{1}$, plans generated using GK‐KBP‐OptIso had slightly lower average conformity compared to those generated using GK‐KBP‐FixIso. However, this trade‐off was offset by a significant improvement in average dose falloff, as measured by the gradient index. Additionally, 59% of the targets achieved better conformity, with 72% showing improved gradient indices. These results are particularly encouraging given none of the candidate isocenter locations and KBP plans were refined or iterated upon. Plan quality could likely be further improved with such refinements.

When considering isocenter limits $I_{0}$, $I_{1}$, and $I_{2}$, KBP with isocenter optimization is capable of producing higher quality plans compared to pipelines without isocenter optimization, albeit with a moderate increase in treatment time. In contrast, plans generated under the predicted isocenter limit $I_{\text{pred}}$ show that superior plan quality can be achieved without increasing average treatment duration. These results highlight three key findings regarding isocenter limits. First, GK‐KBP‐OptIso can generate quality plans without any human intervention as planners would not even need to specify isocenter limits – the strong performance of $I_{\text{pred}}$ is particularly encouraging in this regard, as it suggests the possibility of a fully automated pipeline and motivates further exploration and refinement of the isocenter limit prediction methodology. Second, the isocenter limit and candidate locations impact both plan quality and treatment duration. Finally, while manually chosen isocenters associated with the corresponding clinical plan produced high‐quality KBP results, they are not optimal for usage in a KBP context. Dedicated isocenter optimization is essential to ensure chosen isocenters are optimal in the KBP framework.

While our results demonstrate that increasing isocenter counts can improve plan quality without substantially increasing beam‐on‐time, there are several relevant considerations to note. Our inverse planning model does not account for the time required to transition between isocenters (typically 6–10s),^2^ which can become relevant at higher isocenter counts. In our study, when accounting for this transition time, our best plans required only a marginally longer adjustment time compared to the clinical plans (59 vs. 47 s). Higher isocenter counts can also make plan delivery more difficult. For instance, planners have a lower degree of control over a plan with high isocenter counts as it is more difficult to efficiently review the plan prior to delivery. Additionally, more isocenters can increase the risk of unaccounted dose delivery due to sector motion dynamics.^2^

In principle, every voxel in the target could be a candidate isocenter. However, computational constraints limit the candidate set. We used a closed‐packing of equal sized spheres to generate spatially uniform isocenter locations throughout target volumes. While this ensures systematic coverage of the target, it is not guaranteed to be optimal, particularly for irregular or concave targets where a nonuniform candidate distribution may be more appropriate. For larger, more complex targets, greater isocenter density may be needed along the periphery to ensure all dose is localized within the target. Similarly, for smaller targets, equidistant spacing of candidates may be suboptimal with more restrictive isocenter limits, as none of the candidate locations may provide sufficient coverage for the target alone. To assess an alternative candidate selection approach, we also tested the usage of a hybrid grassfire and sphere‐packing algorithm, which theoretically should produce better results for irregularly shaped targets. However, when tested across all targets, this algorithm resulted in plans of slightly lower average quality. Better methods of choosing candidate locations may lead to more optimal isocenter configurations. These methods may include established approaches, such skeletonization or neural network‐based selection,^24^ heuristics that account for variations in target size and shape,^6^ or adaptive strategies that stratify targets by shape and apply different methods accordingly.

Regarding the choice of weights in the objective function, plans produced using weights derived from the cutting plane algorithm showed no significant differences compared to those generated using weights derived from the approximate formulation (2). Although we observed an average relative gap of 5.3% in the inverse planning objective value, consistent with what has been previously reported,^21^ this gap did not manifest into measurable differences in deliverable plan quality. This is likely due to two factors. First, the dose predictions may not be of a quality where the optimization stage benefits from more precise weight estimations. Second, the relative difference is distributed across all optimization objectives, which themselves only represent approximations of the plan evaluation metrics,^8^ minimizing the overall impact. This is a significant practical finding: the relaxed formulation produces plans of comparable quality to the full cutting plane approach while significantly improving computational efficiency. The results therefore suggest that using weights approximated from the inverse of the linearly relaxed planning model (2) would offer the best balance between plan quality and computational efficiency. However, in scenarios where closer adherence to the dose prediction is beneficial, such as in cases with exceptionally high‐quality predictions, the cutting plane algorithm should remain the method of choice given a reasonable computation time.

The current pipeline does not account for OARs. This is primarily driven by data availability, as only 18% of patients in the broader training dataset had OARs in proximity to targets, making OAR sparing difficult to train for in the prediction model. However, incorporating OARs into the pipeline should be straightforward.^12^ In the prediction stage, an OAR mask could be encoded as a tensor and used as an additional input to the prediction model, allowing for output dose predictions to account for nearby critical structures. In the optimization stage, dose to the OAR can be limited through either separate hard constraints or an additional penalty function in the objective. Together, these modifications would enable the pipeline to produce quality plans while limiting dose to OARs.

The methodology presented has several limitations. First, we used volume‐based optimization shells used to guide the optimization process^8^ not accounting for target surface area, and shifted kernels to approximate the true kernels in the optimization process. Second, the mixed‐integer inverse planning formulation requires longer computation times than previous linear approaches. Combined with the iterative nature of the cutting plane algorithm, the computational burden may limit clinical feasibility for large, complex targets. Third, the big‐M constraints in formulation (1) introduce sensitivity to the choice of M. The choice of M has an impact on both solution quality and the computational efficiency.^2^ Finally, due to data availability, the clinical plans used in this study for both pipeline training and clinical comparison were manual plans. However, we note that many centers now use inverse planning tools, which have been shown to consistently produce plans of slightly higher quality while maintaining treatment time.^4^ Thus, using inverse plans as clinical comparisons could reduce the quality gap observed between plans produced by our pipeline and clinical reference plans. However, we note that the usage of manual plans ensured a more consistent comparison in this paper, given that the 3D dose prediction model underlying our pipeline was trained using manual plans. Additionally, given the overall similar quality of inverse plans compared to manual plans,^4^ we believe our comparisons remain clinically meaningful. Furthermore, given a sufficiently large dataset of inverse plans, retraining our pipeline on such data could further improve its performance, representing a future direction for this work.

## 5 CONCLUSION

This paper presents a novel knowledge‐based planning pipeline for GK that integrates isocenter selection with beam‐on‐time optimization and dose prediction. Predicting appropriate isocenters can improve plan quality compared to a KBP pipeline that uses clinical isocenters. This approach has the potential to streamline the effort required to generate clinical‐quality treatment plans, without compromising quality.

## CONFLICT OF INTEREST STATEMENT

The authors declare no conflicts of interest.

## References

[1] Faramand A , Lunsford D . Gamma Knife radiosurgery: a review of epidemiology and clinical practice. 2020.

[2] Cevik M , Shirvani Ghomi P , Aleman D , et al. Modeling and comparison of alternative approaches for sector duration optimization in a dedicated radiosurgery system. Phys Med Biol. 2018;63:155009. 29972141 10.1088/1361-6560/aad105

[3] Wang T , Giles MD , Butker E , et al. A plan quality control method of treatment planning for Gamma Knife radiosurgery. 2020.

[4] Wieczorek DJ , Kotecha R , Hall MD , et al. Systematic evaluation and plan quality assessment of the Leksell® Gamma Knife® Lightning dose optimizer. Med Dosim. 2022;47:70‐78. 34696931 10.1016/j.meddos.2021.08.006

[5] Perks JR , El‐Hamri K , Blackburn TPD , Plowman PN . Comparison of radiosurgery planning modalities for acoustic neuroma with regard to conformity and mean target dose. Stereotact Funct Neurosurg. 2005;83:165‐171. 16319520 10.1159/000089987

[6] Doudareva E , Ghobadi K , Aleman DM , Ruschin M , Jaffray DA . Skeletonization for isocentre selection in Gamma Knife® Perfexion®. TOP. 2015;23:369‐385.

[7] Ghobadi K , Ghaffari HR , Aleman DM , Jaffray DA , Ruschin M . Automated treatment planning for a dedicated multi‐source intracranial radiosurgery treatment unit using projected gradient and grassfire algorithms. Med Phys. 2012;39:3134‐3141. 22755698 10.1118/1.4709603

[8] Sjölund J , Riad S , Hennix M , Nordström H . A linear programming approach to inverse planning in Gamma Knife radiosurgery. Med Phys. 2019;46:1533‐1544. 30746722 10.1002/mp.13440 PMC6850474

[9] Lee YC , Wieczorek DJ , Chaswal V , et al. A study on inter‐planner plan quality variability using a manual planning‐ or lightning dose optimizer‐approach for single brain lesions treated with the Gamma Knife® Icon®. J Appl Clin Med Phys. 2023;24:e14088. 37415385 10.1002/acm2.14088 PMC10647977

[10] Babier A , Mahmood R , McNiven AL , Diamant A , Chan TCY . Knowledge‐based automated planning with three‐dimensional generative adversarial networks. Med Phys. 2020;47:297‐306. 31675444 10.1002/mp.13896

[11] Babier A , et al. OpenKBP‐Opt: an international and reproducible evaluation of 76 knowledge‐based planning pipelines. Phys Med Biol. 2022;67:185012. 10.1088/1361-6560/ac8044 PMC10696540 36093921

[12] Zhang B , Babier A , Ruschin M , Chan TCY . Knowledge‐based planning for Gamma Knife. Med Phys. 2024;51:3207‐3219. 38598107 10.1002/mp.17058

[13] Zhang B , Babier A , Chan TCY , Ruschin M . 3D dose prediction for Gamma Knife radiosurgery using deep learning and data modification. Phys Med. 2023;106:102533. 36724551 10.1016/j.ejmp.2023.102533

[14] Hoffmann L , Knap MM , Alber M , Møller DS . Optimal beam angle selection and knowledge‐based planning significantly reduces radiotherapy dose to organs at risk for lung cancer patients. Acta Oncol. 2021;60:293‐299. 33306422 10.1080/0284186X.2020.1856409

[15] Chan TCY , Mahmood R , Zhu IY . Inverse optimization: theory and applications. Oper Res. 2023;73:1046‐1074.

[16] Osmancikova P , Novotny J , Solc J , Pipek J . Comparison of the convolution algorithm with TMR10 for Leksell Gamma Knife and dosimetric verification with radiochromic gel dosimeter. J Appl Clin Med Phys. 2018;19:138‐144. 29226607 10.1002/acm2.12238 PMC7663980

[17] Wang L . Cutting plane algorithms for the inverse mixed integer linear programming problem. Oper Res Lett. 2009;37:114‐116.

[18] Bodur M , Chan TCY , Zhu IY . Inverse mixed integer optimization: polyhedral insights and trust region methods. INFORMS J Comput. 2022;34:1471‐1488.

[19] Chan TCY , Lee T , Terekhov D . Inverse optimization: closed‐form solutions, geometry, and goodness of fit. Manag Sci. 2019;65:1115‐1135.

[20] Chan TCY , Craig T , Lee T , Sharpe MB . Generalized inverse multiobjective optimization with application to cancer therapy. Oper Res. 2014;62:680‐695.

[21] Moghaddass M , Terekhov D . Inverse integer optimization with an imperfect observation. Oper Res Lett. 2020;48:763‐769.

[22] Gurobi Optimization, LLC. Gurobi optimizer reference manual. 2024.

[23] Torrens M , Chung C , Chung HT , et al. Standardization of terminology in stereotactic radiosurgery: report from the standardization committee of the International Leksell Gamma Knife Society: special topic. J Neurosurg. 2014;121(Suppl):2‐15. 10.3171/2014.7.GKS141199 25587587

[24] Berdyshev A , Cevik M , Aleman D , et al. Knowledge‐based isocenter selection in radiosurgery planning. Med Phys. 2020;47:3913‐3927. 32473064 10.1002/mp.14305

